# Application of Medical Imaging and 3D Printing Technology in Teaching the Handling of Novel Medicine in Periodontal Surgery

**DOI:** 10.7759/cureus.29271

**Published:** 2022-09-17

**Authors:** Ogawa Tomohisa, Takashi Kamio, Yuuki Maeda, Kento Tsubosaki, Tomotaka Kato, Hiroshi Iwata

**Affiliations:** 1 Division of General Dentistry, Nippon Dental University Hospital, Tokyo, JPN; 2 Oral and Maxillofacial Radiology, Nippon Dental University, Tokyo, JPN; 3 Division of General Dentistry, Nippon Dental University, Tokyo, JPN; 4 Division of Oral Diagnosis, Oral and Maxillofacial Radiology and Pathology Diagnostic Services, Nippon Dental University Hospital, Tokyo, JPN

**Keywords:** surgical simulation, periodontal surgery, medical education and training, fibroblast growth factor (fgf-2), 3d modeling

## Abstract

Recently, fibroblast growth factor-2 (FGF-2) agents for periodontal tissue regeneration have been increasingly applied to the treatment of periodontal disease. Our current challenge for resident dentists with little clinical experience is to enhance instruction in the handling of new medicine in addition to teaching conventional procedures in periodontal tissue regeneration. This report describes using case-specific, cost-effective three-dimensional (3D) models for dentists' lectures and periodontal surgical training.

As an educational and training aid, preoperative and postoperative cone-beam computed tomography images were superimposed to enable three-dimensional observation of postoperative bone regeneration. A three-dimensional anatomical model was fabricated based on these images. Dental laboratory materials were used to reproduce the periosteum and gum. The fabrication time per 3D model was about 2 hours and the cost per model was about $0.5. These models were used for lectures to resident dentists and periodontal surgery training, and their feedback was obtained. The resident's response to surgical training using these 3D models was generally positive.

The use of FGF-2 represents a new direction in the treatment of periodontal disease. This being new, however, means that inexperienced periodontists require training in its application and how this will affect prognosis, as this will differ from that with more conventional techniques aimed at tissue regeneration. The low-cost 3D model presented in this report can be a valuable tool to help accomplish this in teaching inexperienced dentists, such as resident dentists.

## Introduction

The arrival on the market of a fibroblast growth factor-2 (FGF-2) medicine late in 2016 opened up a new direction in therapy aimed at the regeneration of periodontal tissue [1]. It is still considered new today, and many dentists lack knowledge and experience in its use. Getting a lecture on this new drug from a periodontist is one of the solutions to knowing them.

Treatment of periodontal disease is often more time-consuming than other treatment modalities, and periodontal tissue regeneration, which is primarily aimed at regenerating the alveolar bone around the teeth, often requires several years. In addition, even with FGF-2, periodontal tissue regeneration is not an overnight process. Resident dentists are expected to acquire the conventional knowledge and skills for conducting periodontal surgery in a relatively short period, that is, within a limited training period. On top of this, now they must also learn about FGF-2 and its effects. The senior staff must teach them what is required during the long process of treatment, from planning to prognosis. They are also required to provide young resident dentists with opportunities for training in the surgical techniques that such therapy will entail.

At our hospital, periodontal surgical training has traditionally provided the aid of ready-made, commercially available training models. Recent advances and the spread of three-dimensional (3D) imaging and 3D printing technologies have made such surgical training exercises more practical and cost-effective [2,3]. In this report, we present our lectures and surgical training with case-specific 3D models based on medical imaging technology for trainees learning periodontal tissue regeneration using FGF-2.

## Technical report

Case summary as teaching material

One completed case of periodontal tissue regeneration therapy with good results was used as an instructional model. The following is an overview of that case.

A 75-year-old man presented in October 2017 with the chief complaint of gingival bleeding and looseness of the teeth. He was a non-smoker and had no systemic or family history of the disease. His O'Leary Index (plaque control record) score was 61.0%, and protrusion at #16, #27, #37, and #46 was 2 degrees. The mean probing depth (PD) was 4.1 mm, with nine teeth showing a PD of 6 mm or greater. Based on these findings, following the diagnostic criteria of Tonetti et al. [4], the diagnosis was localized periodontitis, stage Ⅲ, grade B.

**Figure 1:**
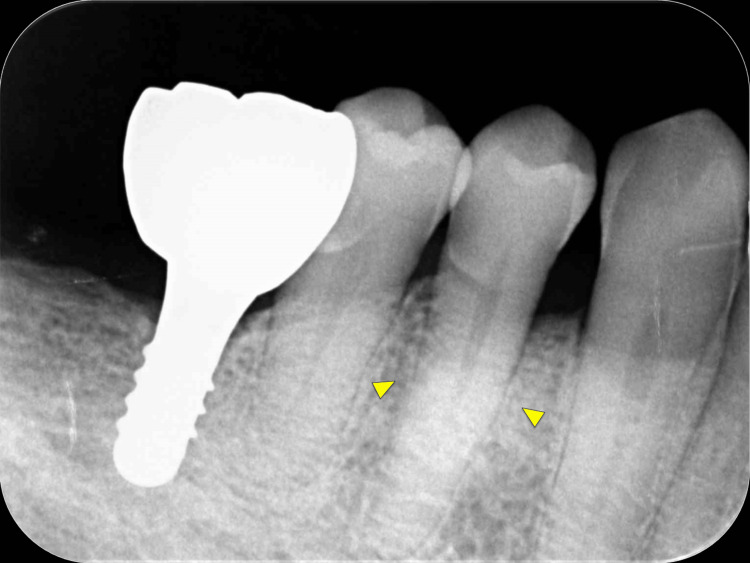
Preoperative intraoral radiographic image. It is difficult to evaluate the condition of the alveolar bone defect around tooth #44 on the intraoral radiographic image (arrowheads).

**Figure 2:**
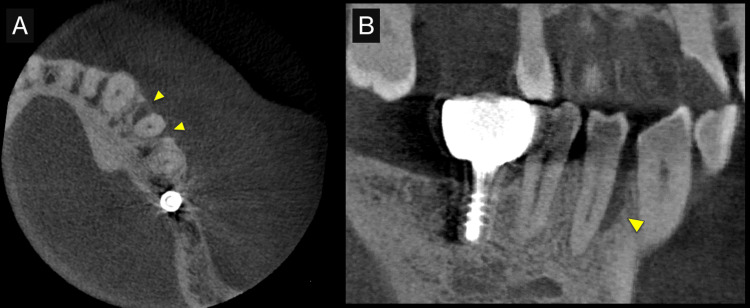
Preoperative cone-beam computed tomography (CBCT) images. (A) CBCT axial view, and (B) CBCT oblique sagittal view. CBCT images allowed the detection of the defect in 3D (arrowheads).

First, initial treatment including oral hygiene instruction was provided, followed by scaling and root planing. Teeth #16, #27, #37, and #46 were extracted, as they were considered too difficult to preserve. After reevaluation, a dental implant was provided for tooth #46. The patient was enrolled in supportive periodontal therapy after it was confirmed that the initial course of periodontal treatment had stabilized conditions. Subsequently, an acute periodontal abscess was observed in #44, however, requiring the start of anti-inflammatory treatment including periodontal pocket curettage under local anesthesia and oral antibiotics. Nevertheless, no improvement was found in the 6-mm buccal pocket of #44, so periodontal tissue regeneration therapy was planned. Before starting such treatment, in addition to intraoral radiography, cone-beam computed tomography (CBCT) was also performed using the 3DX Multi Image Micro CT (J MORITA Co., Ltd., Kyoto, Japan) (Figures 1, 2). The scan parameters were as follows: 90-kV tube voltage; 7-mA tube current; field of view, 60 mm x 60 mm; and slice thickness, 0.125 mm. The CBCT images revealed an intrabony, two-walled, defect around #44 that was slightly less radiolucent on the intraoral radiographic image. It was localized on the buccal side from the proximal to the distal aspect. A relatively large mandibular torus interior was also observed on the lingual side of #44 and #43.

Periodontal tissue regeneration therapy was selected as the surgical procedure to be used considering the maintenance of blood supply from the surrounding tissues as a priority. Prior to surgery, a 3D model was fabricated based on the preoperative CBCT images using a 3D printer.

**Figure 3:**
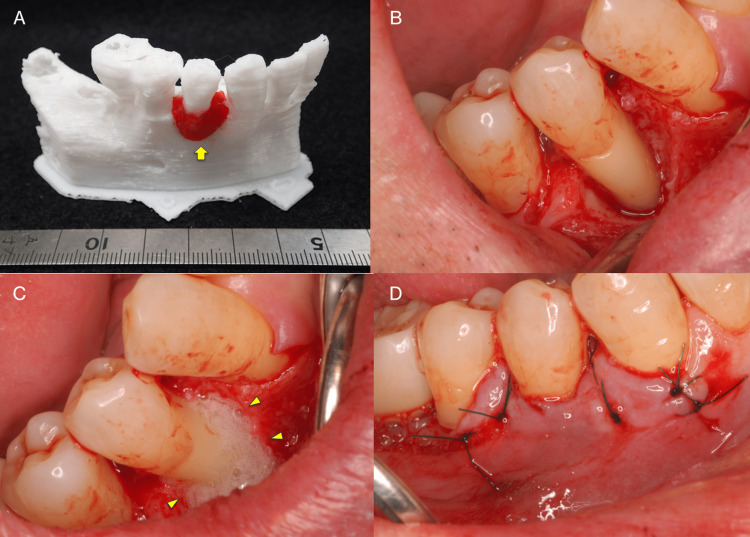
3D model used for the FDF-2 medicine applicative simulation and photographs during periodontal surgery. (A) The area to which the medicine is to be applied is shown with red utility wax (arrow). (B) A mucoperiosteal flap was dissected to define the surgical site. (C) REGROTH® was applied to the intrabony defect (arrowhead). (D) The area was sutured tightly to prevent leakage.

The fabricated 3D model was then explained to the patient and a simulation was run to confirm the planned use of FGF-2 and the required amount is shown in Figure 3A. Photographs taken during the surgery are shown in Figure 3B-3D. The surgical procedure was performed under local anesthesia (2% lidocaine with 1:100,000 epinephrine). Just one incision was made on the buccal side of the gingival sulcus, and a simplified papilla preservation technique was employed to preserve a wide margin between the mucoperiosteal flaps at the interdental papilla incision [5]. The dissection to reveal the intrabony defect was kept to a minimum to maintain blood flow, with only the buccally attached gingiva being incised. A full-thickness mucoperiosteal flap was then developed. After completion of debridement, a relatively large intrabony defect was observed on the buccal side of #44 spanning the proximal to the distal aspects. Periodontal regenerative medicine (REGROTH®, Kaken Pharmaceutical Co., Ltd., Tokyo, Japan) was applied to the defect, and the flap was closed. It was also injected into the gap during suturing to avoid a decrease in volume due to leakage. After surgery, the patient was instructed to use a mouth rinse containing chlorhexidine (ConCool F®, Weltec Corp., Osaka, Japan) regularly for four weeks. No significant irregularities were observed postoperatively (no excessive swelling, and no suture breakage or infection) and the sutures were removed one week later. After that, the patient was instructed to brush with a soft-bristle toothbrush to prevent damage to the treated area. Brushing using the scrubbing method was started in the second week postoperatively. Meanwhile, the patient continued to receive constant professional care.

**Figure 4:**
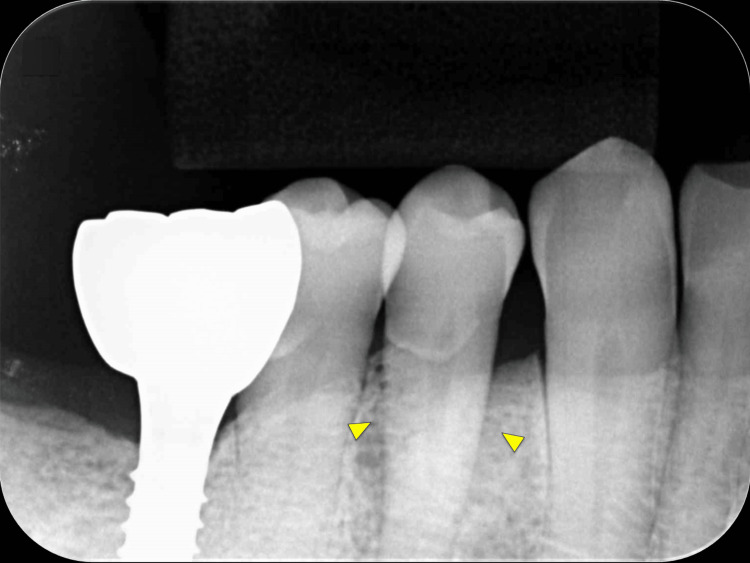
Postoperative intraoral radiographic image. Comparing only the intraoral radiographic images preoperatively and postoperatively, little change was seen in the radiolucency of the alveolar bone around tooth #44 (arrowheads).

**Figure 5:**
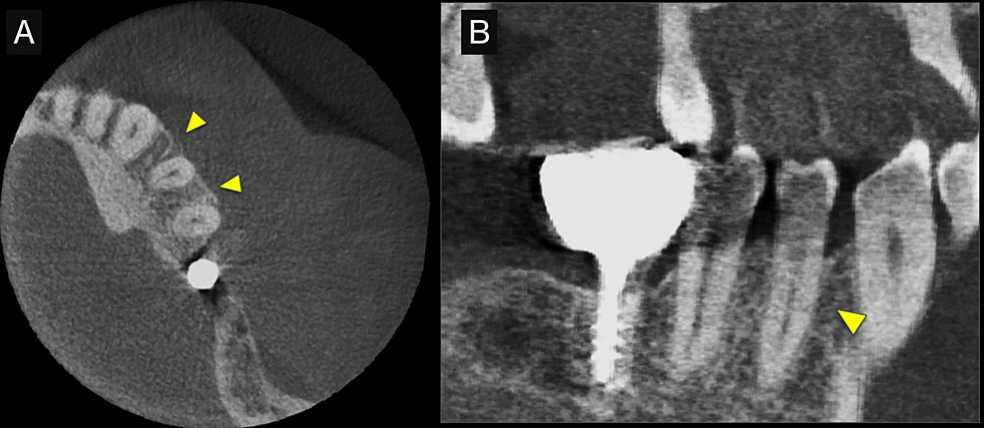
Postoperative cone-beam computed tomography (CBCT) images. (A) CBCT axial view, and (B) CBCT oblique sagittal view. CBCT images showed the bone-like structures formed in the intrabony defect area (arrowheads).

Intraoral radiography and CBCT scanning were performed 14 months postoperatively (Figures 4, 5). The results were displayed on a personal computer monitor screen, and the morphology of the bone at the surgical site and its internal structure was observed in 3D, by which the regrowth of bone-like structures at the site of the intrabony defect was confirmed. At two years postoperatively, no subjective symptoms such as pain or objective signs such as tooth movement or gingival recession were observed. No other notable findings or abnormalities were observed, and the postoperative course was judged to be good.

**Figure 6:**
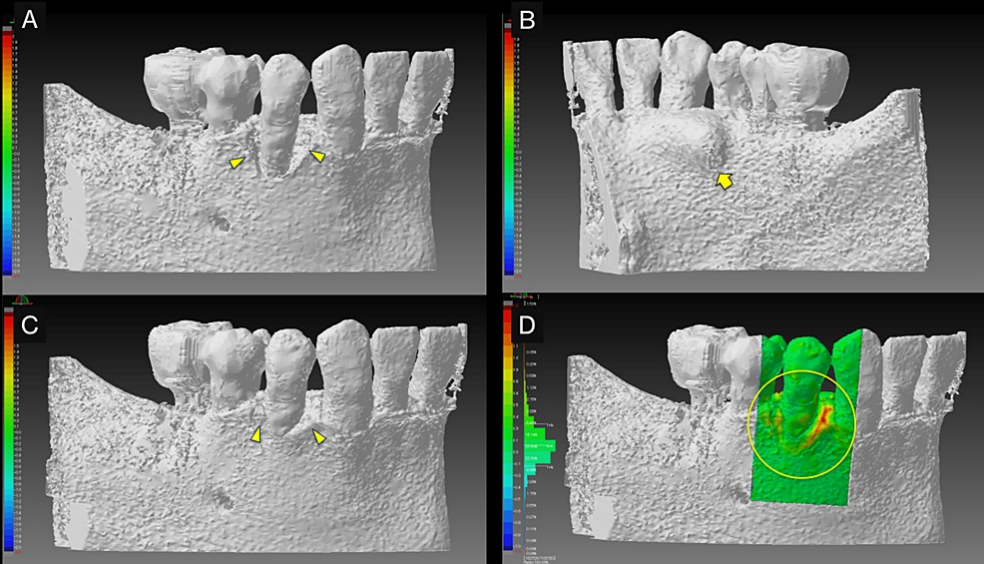
3D views of pre- and post-operative cone-beam computed tomography (CBCT) images and superimposed images of pre- and post-operative CBCT images. (A) Preoperative CBCT images show intrabony defects (arrowheads). (B) A mandibular torus on the lingual side of tooth #44, would have made it difficult to assess the intrabony defect on the intraoral radiographic image (arrow). (C) CBCT image at 14 months postoperatively shows a bone-like structure in the intrabony defect on the buccal side of tooth #44. (D) In the preoperative-postoperative superimposition, areas suggestive of bone regeneration are shown in yellow to red (circle).

In teaching a clinical case, it is necessary to clarify the treatment goals. Therefore, polygonal mesh models were created from superimposed pre- and postoperative CBCT images to allow 3D observation of therapeutic changes during periodontal tissue regeneration (Figure 6). The 3D computer-aided design (CAD) inspection software package "SpGauge 2014.1" (Armonicos, Shizuoka, Japan) was used to display dimensional errors on a color map.

3D models for surgical training

**Figure 7:**
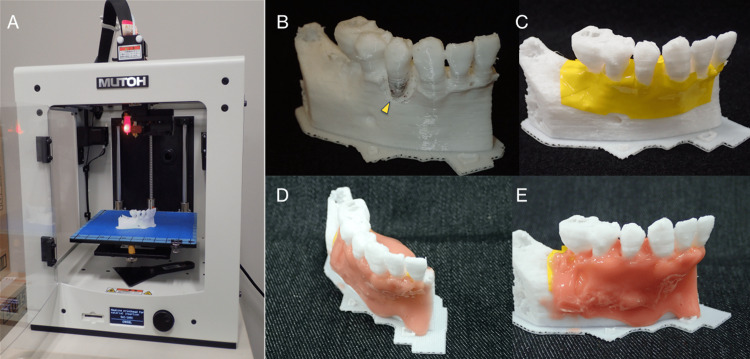
3D printer and fabricated 3D models. (A) Desktop FDM 3D printer Value3DMagiX MF-800. (B) A 3D model with colored tooth root to explain to the patient and indicate the root-planing area (arrow). (C) A 3D model with yellow vinyl tape to simulate the periosteum. (D, E) The 3D model with gum applied.

The polygonal mesh models were created from Digital Imaging and Communications in Medicine (DICOM) images in STL format data using the medical image-processing software package Volume Extractor 3.0 (i-Plants Systems, Iwate, Japan) [6]. These data were based on the preoperative CBCT images. First, slicing software was used to generate control data of the 3D printer (G-code data). Three-dimensional anatomical osseous models of the teeth and jawbone were then fabricated using the desktop fused deposition modeling (FDM) 3D printer Value3DMagiX MF-800 (MUTOH Industries Ltd., Tokyo, Japan) (Figure 7A). The 3D printing parameters were as follows: filament used, 1.75-mm; polylactic acid resin (PLA) (Pxmalion, eTranslab Inc., New York, USA); lamination pitch, 0.2 mm; infill density, 33%; printing speed, 40 mm/sec; heated nozzle temperature, 210℃; without support structure or raft. To apply soft tissue to the teeth and jawbone in the 3D models fabricated with the 3D printer, the colored vinyl tape was pressed in to imitate the periosteum and then a silicone rubber-based impression material (Multisil-Mask Soft, Bredent GmbH & Co. KG, Senden, Germany) was applied manually to simulate the gum (Figure 7B-7E).

Although the slicing software (Cura customized for MUTOH, MUTOH Industries Ltd., Tokyo, Japan) calculated that the fabrication time for a 3D model would be about 2 h, the 3D printer used allowed the fabrication of five 3D models at a time, so altogether it took around 8 h. The actual cost of one teeth and jawbone 3D model for periodontal surgical training fabricated with the filament used (24 USD/1000 grams) was approximately 0.6 USD, including the cost of a few grams of silicone rubber-based impression material for the gum.

**Figure 8:**
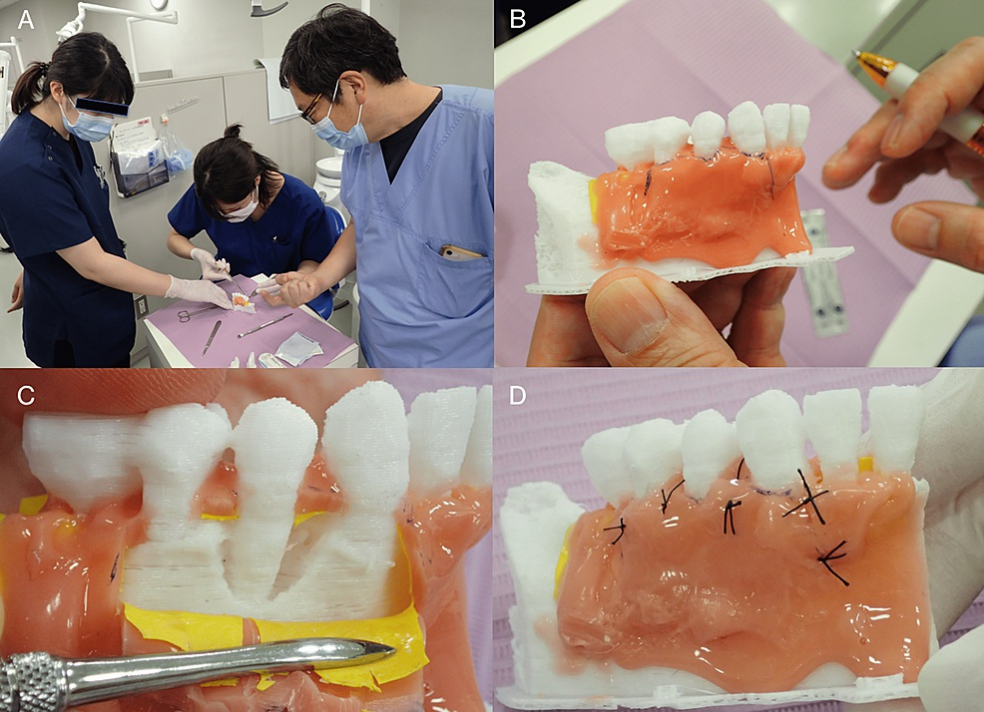
Surgical training using 3D model. (A) The 3D model itself is light and requires fixation by an assistant. (B) Marking of the incision line. (C) Dissection of the flap. (D) Flap closure and suturing.

The surgical training shown in Figure 8 represents our newly proposed method for such, not a pre-packaged training course for resident dentists. Open-ended responses were obtained from the four resident dentists and two instructors who participated in this surgical training. Resident dentists' feedback and comments are shown in Table 1.

**Table 1:** List of comments by resident dentists (with multiple responses).

Resident dentist's comments	Number of the resident dentist
The incision line and extent of flap dissection could be clearly identified. It was clear to me how much of the flap needed to be detached to ensure that the surgical site was clearly visible.	4
Periosteal dissection must be done carefully or the flap will detach. I understood that the interdental papillae had to be treated carefully.	3
Since the 3D model is made of the same material in a single color, it would be more realistic if the teeth and bones were of different hardness and color.	2
I wish I could have installed a 3D model on the practice phantom. The 3D model is a bit light, it needs to have more weight, like a ready-made model.	1
The flap on the 3D model is a bit more fragile than a real flap, so it needs to be more ductile.	1
Utility wax was used to show the amount of the drug to be filled into the intrabony defect but to make it more realistic, it would be better to find a material with properties similar to those of the actual FGF-2 drug (a material with better flowability).	1

## Discussion

The history of periodontal tissue regeneration therapy is long, beginning with Hegedus' report of autogenous bone grafting in 1923 [7]. Approximately 100 years later, in December 2016, the world's first periodontal tissue regenerator, REGROTH®, which contains recombinant human basic FGF-2 as its active ingredient, was launched. The use of FGF-2 represents a paradigm shift in periodontal tissue regeneration therapy from guided tissue regeneration (GTR), enamel matrix protein (EMD) application, and bone grafting [8,9]. Today, numerous periodontists have reported on the indications for, the clinical outcome of, and specifics of various procedures involving the use of FGF-2 [1, 10-12]. In Japan, the use of FGF-2 in periodontal surgery is predicted to become more widespread now, it is available under the national health insurance system. Fibroblast growth factor-2 may only be used in periodontal surgery if it conforms to a set of basic rules concerning efficacy. Moreover, this must be done in strict compliance with the guidelines concerning dosage and administration. Its use is indicated in cases where there is a vertical bone defect with a periodontal pocket depth of more than 4 mm and a bone defect depth of more than 3 mm in reevaluation following the completion of basic periodontal treatment. It may only be used by a dentist or physician with experience in periodontal surgery and should be applied to the alveolar bone defect at the time of gingival debridement curettage.

In learning about periodontal surgery, an inexperienced resident dentist must master the necessary conventional techniques through training in procedures such as GTR, application of EMD, and bone grafting. They are then required to master more advanced options such as the use of FGF-2, which necessitates learning about the characteristics [13]. The job of teaching staff in this respect is to impart such knowledge and the relevant skills, whether it be for use of FGF-2 or more conventional procedures. Ready-made training models have long been used in periodontal surgical training. Because these models are standardized, their form is naturally not specific to the individual patients that periodontists encounter in their clinical practice. Additionally, they are expensive, so the same model is usually used repeatedly.

Conventional images illustrate only two-dimensional images of three-dimensional structures [14] and 3D construction will help in the clear establishment of surrounding structures [15]. And an anatomical craniofacial 3D model of them in three dimensions has long been used to simulate oral surgery [3,16,17]. Recently, there have been an increasing number of reports on the use of 3D models that reflect real patient situations in dental education [18]. To our knowledge, although there have been reports of the use of 3D models in general dental practice for surgical endodontics and tooth autotransplantation [19], there have been no such reports for training on the use of FDM 3D printers and case-specific 3D models in periodontal treatment.

By using this 3D model, we could see and touch the condition of the defect to be treated and thus know the amount of FGF-2 to use before the application. The main advantage of the FDM 3D printing system is its economic efficiency [20]. The availability of low-cost 3D models should increase the number of training opportunities. The key point here is that the 3D models that are fabricated are not those of typical cases, like those reflected in ready-made training models. Rather, they are custom-made and so faithfully replicate the specific conditions encountered in each patient.

However, because it is a low-end/personal desktop FDM 3D printer, it is not suitable for mass fabrication of 3D models in a short time. It is difficult to fabricate enough 3D models for surgical training when many trainees are to be taught on the same occasion. Moreover, there are also problems related to the materials to be used. Usually, only monochromatic and relatively hard materials such as PLA resin can be used presently, making it difficult to fabricate 3D models that reproduce soft tissues such as gum. It is hoped that softer materials will be available in the future that can reproduce soft tissues.

## Conclusions

This report described the development of the 3D printed model for simulating periodontal surgery, and the feedback and opinions sought from the resident dentists who were trained using this 3D model. The case-specific 3D models are a cost-effective tool for more efficient teaching by using them as a tool to assist in the acquisition of surgical skills. Learning practical surgical techniques through 3D models fabricated with this advanced technology can benefit all dentists, not just students, and resident dentists.
